# The Collateral Damage of the COVID-19 Outbreak on Mental Health and Psychiatry

**DOI:** 10.3390/ijerph18094440

**Published:** 2021-04-22

**Authors:** Frederick A. J. Simon, Maria Schenk, Denise Palm, Frank Faltraco, Johannes Thome

**Affiliations:** Clinic and Polyclinic for Psychiatry and Psychotherapy, University of Rostock, 18147 Rostock, Germany; Maria.Stuth@med.uni-rostock.de (M.S.); Denise.Palm@med.uni-rostock.de (D.P.); Frank.Faltraco@med.uni-rostock.de (F.F.); Johannes.Thome@med.uni-rostock.de (J.T.)

**Keywords:** psychiatry, SARS-CoV-2, COVID-19, stigma, discrimination, disparities

## Abstract

The potential consequences of the COVID-19 outbreak are multifarious and remain largely unknown. Deaths as a direct result of the condition are already in the millions, and the number of indirect deaths is likely to be even higher. Pre-existing historical inequalities are compounded by the virus, driving increased rates of infection and deaths amongst people who use drugs and alcohol, those belonging to racial-ethnic minority groups, poorer communities, LBGTQ+ populations, healthcare workers, and other members of the care economy; all of whom are already at increased risk of adverse mental health effects. In this paper we suggest that a central role of mental health practitioners is advocacy: both for people who use psychiatric services and for those who, due to the effects of the pandemic, are at an increased risk of needing to do so.

## 1. Introduction

Although the severe acute respiratory syndrome coronavirus 2 (SARS-CoV-2) is only 105 nm in diameter, it casts a long shadow. Historical inequities continue to drive unbalanced rates of COVID-19 infection [1]. For example, all-cause mortality amongst those from black and minority ethnic (BAME) communities is almost four times higher than amongst those from white ethnic groups [2]. Those raised in poverty are be more likely to die from COVID-19, as are those known to psychiatric services [1]. The risk factors are diverse and not yet clearly understood, but seem to include obesity, increased rates of exposure to the virus, the cumulative presence of co-morbid illness, and potential difficulties in following public health protection measures [3,4].

The effect of the pandemic reverberates beyond the direct deaths as a result of the infection. The current number of those vaccinated (363,691,238) outnumbers confirmed cases (120,164,106) by a factor of three [5]. Despite the mass production of efficacious vaccinations, infection rates remain high. In addition, the outbreak has affected mental health service provision: outpatients clinics have been cancelled, inpatient services curtailed, postponement of treatment is widespread, and community support for service users with ongoing needs has been limited in many regions worldwide [6,7,8,9]. In a survey of its 130 member states, the WHO found that most countries had experienced disruption to mental health services. While 30% of countries were forced to redeploy mental health workers to the COVID-19 response, 19% had to repurpose psychiatric facilities, and 89% suffered shortages of PPE amongst mental health workers [10]. Moreover the mental health consequences of non-pharmaceutical interventions (NPIs), including government lockdown, physical distancing, isolation, and an economic recession can already be felt and are likely to only become more pronounced [11,12,13,14,15]. This work summarizes (1) the direct impact of COVID-19 on people who use mental health services, and (2) the effect on those with an increased risk of needing to.

For those already in treatment, or who have previously used mental health services, we discuss the risks associated with the closure of outpatient psychiatric services, the limitations and restrictions on inpatient services, the specific impact on particular patient groups, and barriers to seeking treatment. For those not known to mental health services, we focus on the impact of the COVID-19 outbreak on women’s mental health, health care professionals, people who use drugs and alcohol, and the vulnerably employed and those at risk of involuntary redundancy [10,16,17,18].

## 2. Effect of COVID-19 on People Who Use Psychiatric Services

### 2.1. Reduction of In-Person Outpatient Psychiatric Services

Governments globally have focused their efforts on minimizing deaths as a result of COVID-19. This has been largely enacted through measures designed to reduce or stop transmission. In Italy, one of the first European countries to be affected, widespread closures of mental health services were enforced. In order to maintain essential functions, direct patient encounters in second- and third-level outpatient units (eating disorders, psychiatry for older people, adult neuropsychiatry, ADHD, and adult autism) were replaced with telephone calls and video conferences. General outpatient services were restricted to include just those requiring urgent visits and daily administration of medicines or long-acting injectables [6].

For those with chronic conditions such as schizophrenic psychoses and other long-term mental-health issues, who receive the majority of their care in an outpatient setting, the consequences may be stark. Each in-person appointment increases the transmission risk for both patients and providers. However reductions in these services, designed to protect service users from infection, could have the opposite effect, increasing the risk of service disengagement, medication non-adherence, and psychic distress; all leading to possible decompensation, higher levels of positive symptom severity, and relapse [8,19].

Much of what was previously offered as an outpatient service is now offered through telemedicine. The provision of telehealth has the dual advantages of allowing clinicians to work from home, where they can care for dependents, whilst simultaneously reducing the risk of transmission [1]. It does, however, have serious shortcomings, which are still only beginning to be understood. This can be illustrated by the utilization of telehealth in the context of outpatient eating disorder services. The COVID-19 outbreak has seriously disrupted the treatment of those with eating disorders in the UK, causing population movement, such as young people returning to their childhood home, and having to cede control of cooking and food intake, as well as disrupting strict meal planning and monitoring of calorific intake. Telemedicine is frequently cited as a suitable and necessary replacement of face-to-face interactions [1,6,9]. However interactions of this type can be particularly problematic when treating eating disorders. Service users report wanting to be able to see the person they are talking to, but also not wanting to see themselves [7]. This is indicative of the many problems related to video-conferencing. It is convenient and can be used effectively in an emergency, but important aspects of the therapeutic relationship, such as body-language and eye contact, are lost.

### 2.2. Limitations on Inpatient Psychiatric Assessment and Treatment

The example of just two large outbreaks of COVID-19 in psychiatric units, in Korea (100 of 102 service users in a single unit infected) and China (50 services users and 30 staff in Wuhan Mental Health Centre), indicates the potential scale of the current challenges to inpatient psychiatric care; these are not limited to reducing infection, the extent of service provision, and quality of care is also affected [8].

Much of the pharmacological treatment used for those with pre-existing psychiatric illness interacts with those used against SARS CoV-2. Through the inhibition of CYP450 3A4, some antiretrovirals (atazanavir and liponavir/ritonavir) can significantly increase the levels of antipsychotic medications (including quetiapine, ziprasidone, and pimozide) and benzodiazepines (midazolam and triazolam). Carbamazepine, however, will inhibit the activity of anazanavir, chloroquine, and hydroxychloroquine through induction of CYP3A4 [9].

For those already receiving inpatient psychiatric treatment, the impact has been profound. Anecdotally, discharges have been delayed due to reductions in community based services, access to psychotherapy has been curtailed to reduce transmission rates between wards, and psychiatrists have often been seconded to other medical specialties, leaving a shortage in their own clinics.

Much of the treatment that was previously thought to require hospitalization has been delivered in the community. Driven by necessity, interventions such as the administering of long term injectables, regular blood count measurements, and the monitoring of lithium and clozapine levels have been staged beyond the confines of the hospital setting, through projects such as *mental health home care* and *hospitalization at home* [9]. The COVID-19 outbreak has given a sense of urgency to psychiatric reforms, these changes should be assessed as long term alternatives to standard care in the post-corona-era.

### 2.3. Avoidance Behaviour

Those most susceptible to the lasting impact of a disaster or pandemic are also those most likely to lack direct access to mental health services. Vulnerable populations, such as older patients and those from a lower socioeconomic background are least likely to seek medical attention for both physical and mental health concerns [20].

Fear of being infected, fear of infecting others, worries surrounding loss of livelihood, or being forcibly isolated are all barriers to seeking psychiatric help in the time of a pandemic. High quality information about when to seek help is essential to counteracting these obstacles to care, as is coherent and consistent public health information. This is particularly evident in cases of those in suicidal crises, which is discussed below in more detail [21].

Those with obsessive compulsive disorder (OCD) or OCD symptoms are at an increased risk, particularly sub-groups with concerns about becoming contaminated themselves, individuals with a tendency to seek reassurance by excessive searching for news on COVID-19, people who fear unknowingly spreading contamination and causing harm to others, and people who overestimate threats. Behavioral treatments and targeted information are likely to benefit this population [22].

### 2.4. Impact on Specific Patient Groups

Those in psychiatric treatment face a dual risk. To take those with eating disorders for example, they are often at a higher physical risk (electrolyte disturbance in bulimia nervosa, frailty in anorexia nervosa) as well as being vulnerable to a psychiatric decompensation as a result of the distress and uncertainty introduced by isolation and quarantine [7]. In older patients, this effect is even more profound. In Italy, those aged 70 or older constitute a third of confirmed cases, and make up nine out of ten deaths [3]. In this demographic the prevalence of dementia and depression is also elevated; 11.9% of those who died in Italy had a diagnosis of dementia, making it one of the most common co-morbidities of COVID-19 infection [4]. This may be related to the difficulty of reinforcing public health measures aimed at reducing transmission. Those with a mild cognitive impairment (MCI) or milder dementias may be unable to comply fully with hand washing, maintaining physical distance, covering ones mouth and nose when coughing, self-isolating, and be more prone to wandering [3]. A thorough discussion by EE Brown et al. (2020) considers the consequences that COVID-19 will have on the diagnosis and clinical follow-up of dementia, the disruption to its pharmacological and non-pharmacological management, and the changes to care settings in the community and hospitals during lockdown.

It is too early to predict the consequences of the COVID-19 pandemic for older patients, but following the Hong-Kong SARS epidemic in 2003, elderly suicides significantly increased compared to the consistent reduction in rates seen in the previous two decades [23]. Older patients, who are more susceptible to COVID-19 infections, are also more vulnerable to its consequential biopsychosocial disruption. They have a higher prevalence of pre-existing risk factors for suicide, including a high burden of chronic illness and a lack of social support [23]. Early data in India suggests a worrying trend [24]. Primary health professionals should be particularly aware of the possibility of increased suicide rates in the elderly following the COVID-19 outbreak. 

Those suffering from schizophrenic psychoses are not just more likely to contract COVID-19, they are more likely to die from it; they are more likely to experience a deterioration of their psychiatric symptoms, and they are more likely to misperceive risk and fail to adopt protective measures [25,26,27]. They are particularly vulnerable to the consequences of the pandemic, and rather than have their care funding reduced and redirected to managing COVID-19, they should instead receive increased support. In Italy, those with schizophrenia who cannot access normal levels of care have been contacted to verify full understanding of the government lockdown procedures and to be instructed on basic hygiene requirements [6]. This is an example of the minimum standards that should be expected.

The existing data on the impact of the COVID-19 outbreak on those with preexisting mood disorders is minimal. However there is a broad consensus that those with psychiatric disorders might experience a worsening of symptoms, and thus an increased suicide risk [21]. There will be an increased burden on volunteer run helplines, which will require additional support, as well as on mental health services, which will need to develop clear remote assessment protocols and care pathways for people who are suicidal [21].

Another vulnerable group requiring additional support during isolation are people who use drugs (PWUD), regardless of the presence of a substance use disorder or not. Sharing of drug paraphernalia leads to increased transmission rates in a population that suffers from disproportionately high levels of chronic illness. PWUD suffer regular discrimination and stigmatization, and should be: (1) specifically prompted to seek medical attention when required, (2) encouraged to following social distancing and hand hygiene advice, (3) discouraged from drug and drug-paraphernalia sharing. In addition drug misuse services should be safeguarded, rather than defunded, to protect this already vulnerable population [11].

In Attention Deficit Hyperactivity Disorder (ADHD) and autism spectrum disorders, a negative mood state is associated with increasing symptom severity. Loss of daily routine and a lack of interpersonal and social interactions are all risk factors for a symptomatic deterioration. In children the problem is amplified by parents assuming educational responsibility from specialist teachers [28]. Amongst adults it is possible that the monotony of isolation and the lack of external stimulation may lead to sensation seeking and increasingly risky behavior, resulting in increased infection rates amongst this patient group.

## 3. The Effect of COVID-19 on People Who Do Not Use Psychiatric Services

### 3.1. Impact on Mental Health of Healthcare Providers

Wherever the virus has struck, healthcare providers have been significantly affected. Many have died from the condition, and some have taken their own lives. Shortages of protective equipment have led to understandable fear, and insufficient resources have caused feelings of powerlessness. Staff are under extreme pressure, subject to stigmatization, and are often isolated from friends and family. In a Chinese study, rates of depression amongst healthcare workers were as high as 50%. Anxiety was present in 44% percent of those questioned, with 44% suffering from insomnia, and 71% reporting a subjective feeling of distress [29]. Amongst nurses managing acute cases of coronavirus, 71.4% have reported sleeplessness [30]. The first case of “COVID-19 paranoia” has been published in Germany in the context of chronic schizophrenia, and is unlikely to be an isolated case [31]. These problems may well persist beyond the time limit of the outbreak, and are likely to increase rates of burnout and work-related illnesses. This is compounded by the findings that healthcare workers are more likely to experience bullying in the time of the pandemic. Risk factors for bullying included a perceived loss of respect in the community, and the belief that healthcare workers are the subject of gossip, which increase suffering further. [32]

The interesting concept of “moral injury” has been raised in frontline staff who are required to make treatment decisions regarding intubation and access to ventilation. Organizational support will be central to supporting decision makers in this context to reduce the risks of long-term mental illness [33]. The duration of the psychological impact of treating patients with coronavirus is at this point uncertain, but regular monitoring, screening, and treatment are all likely to become necessary. Comparisons of the impact of the COVID-19 outbreak on frontline staff has been compared to those affected by 9/11. The authors concluded that: “rather than the sudden jolt of fear and horror that accompanied the 9/11 attacks, the COVID-19 pandemic will likely bring a more insidious wave of anxiety, anger, and grief as casualties increase” [34]. With the virus yet to be controlled, its consequences will continue to build.

### 3.2. Psychiatric Sequelae of the Economic Instability, Isolation, Quarantine, and Social Distancing

Almost 800,000 people worldwide die by suicide every year [13]. There are many associated risk factors, including recent unemployment. Data collected after the 2008 financial crisis showed a 20–30% increase in the risk of suicide after becoming unemployment. Estimates predict that worldwide unemployment could increase from 4.936% to 5.644% in the wake of the pandemic [13]. This would equate to 9570 deaths per year. However, each suicide is, on average, associated with 20 suicide attempts and this increased incidence would have a profound impact on psychiatric services. Data from 2008 showed that an increased suicide rate can precede the actual rates of unemployment. To mitigate this effect, information regarding access to mental health services could be targeted to those who have already been furloughed, or are in vulnerable employment [13]. People with salaried jobs are less likely than those on a daily wage to be affected by the instability induced by the pandemic. This will have a substantial and disproportional effect on low income countries [10].

There will be a substantial psychological impact for those with and without pre-existing mental health conditions in the context of significant social and legal restrictions imposed to control viral transmission. Screening for depression, anxiety, and distressing loneliness can be successfully implemented, and actions against these conditions enacted. Amongst Jungian analysts the COVID-19 outbreak has many of the dynamics of the apocalypse archetype. It embodies overwhelming stresses: grief, unemployment, death, isolation, and powerlessness [35]. In an early study of the population of Hong-Kong following the pandemic, authors found 19% of the respondents had depression (PHQ-9 score ≥ 10) and 14% had anxiety (GAD score ≥ 10). These rates are significantly higher than normal background levels. The research team suggested multiple factors influencing poorer outcomes, including government lockdown, and “information overload”. Contradictory information, often prevalent on social media is strongly associated with an increased prevalence of mood disorder symptoms. Frequent social media exposure (SME) was demonstrated to significantly increase the adjusted odds of anxiety compared with less SME after controlling for all covariates (OR = 1.72, 95% CI: 1.31–2.26) [36]. Digital platforms with a firm and growing evidence base can however be utilized in the form of social prescribing, as well as to maintain existing support structures [15]. Psychological sequelae of quarantine and isolation range from feelings of guilt, anger, and hopelessness, to symptoms of post-traumatic stress disorder. Inadequate supplies, inadequate information, fears of infection, and frustration and boredom all worsen the psychological impact of quarantine, whilst stigma and financial stress contribute to poorer outcomes following the easing of restrictions [16]. Mitigations techniques include the supply of clear and reliable information, a short and fixed duration of quarantine, and using voluntary rather than mandatory restrictions when possible [14].

The emergency restrictions to freedom of movement have many inspired anger amongst a diverse range of figures. Increased rates of domestic violence are amongst the most concerning aspects of these protective measures. EU states have reported an increase of up to 60% in emergency calls regarding domestic violence, and there are strong arguments that protection of girls and women must be built into any response plans [12]. At particular risk are older women, women with disabilities, women living in the context of humanitarian crises contexts, poor women living in crowded conditions, and those from ethnic minority communities. Moreover, 30% of women experience sexual or physical violence by an intimate partner in their lifetime, and in periods of acute stress, cramped living conditions, and economic strain, incidence of domestic violence becomes ever higher [12].

Domestic violence is closely associated with problematic drug and alcohol use. Two contradictory models regarding alcohol use and COVID-19 currently exist. The first is an increased rate of use due to distress experienced as a result of the pandemic. The second predicts a lower alcohol consumption based on decrease physical and financial availability of alcohol. It is currently unclear which scenario will dominate, but in the context of current restrictions the second is likely to prevail until the first becomes more relevant in the medium and long-term future [18]. Increased alcohol use has consequences for somatic medicine and psychiatry, both in the treatment of acute detoxification, and the increased incidence co-morbid mental health disorders. Public health reminders of safe drinking limits and messaging about monitoring alcohol intake are low cost interventions that could reduce the direct and collateral damage of alcohol consumption.

The global variation in the response to COVID-19 alludes to the significance of physical geography. In a study using data gathered in Korea, the risk of COVID-19 increased in line with immutable factors, such as higher area morbidity, crowding, and population mobility. Lower rates of social distancing, as well as poorer access to healthcare and education, also increased the risk of infection [37]. These variables can only be challenged using a broad, population based approach to systematic change.

In a far reaching review by Antonio Baldassarre et al. (2020) [38], the significance of stigma and discrimination was critically assessed. Through the introduction of the concept of SAD (stigma and discrimination), the authors explore the implications for both mental and medical health of the people affected by infectious diseases, including COVID-19, as well as creating a framework to challenge the stigmatization of psychiatric illness [38].

### 3.3. Gender Dimensions of COVID-19

Research in Wuhan, suggests a population wide prevalence rate of post-traumatic stress symptoms of 7% one month after the epidemic peaked. However symptoms of re-experiencing, negative alterations in cognition or mood, and hyper-arousal were significantly higher in women than men [17]. 

There are many reasons that women are inequitably affected by the consequences of the COVID-19 outbreak. Economic impacts compound pre-existing inequalities: women are more likely to earn less (16% less) than men, be in insecure employment (740 million women work in the informal economy globally), and live in extreme poverty (25% more women than men). Reallocation of resources away from reproductive and sexual health disproportionally affects women [39]. Simultaneously, women are more likely to be involved in unpaid care work, which is particularly abundant in the context of school closures, and widespread changes in employment structures during the pandemic. They are more often involved in emergency child and elderly care, increasing risk of infection, as well as reducing wellbeing [40]. The additional and disproportionate psychological stressors on women in the wake of COVID-19 increases the risk of developing new psychiatric conditions or experience a deterioration in those already existing. Obscuring sex and gender differences in the wake of the pandemic risks compounding already existing inequalities and addressing the health needs of girls and women directly will help societies recover in a more rapid and more holistic manner [39].

Members of the LGBTQ+ community experience may be at a particular risk of a deterioration in their mental as a result of non-pharmaceutical government interventions when compared to their heterosexual cis-gendered counterparts [41]. The risk is particularly high for those who experience parental rejection or identity concealment in the context of stay-at-home-orders and lockdowns. Amongst LBGTQ+ youth, one third experience familiar rejection, and another third choose not to reveal their gender or sexual identity until they are adults. People who experience familial rejection are six times more likely to develop depression, and eight times more likely to attempt suicide [41]. It is important for mental health professionals to remain aware of the unique challenges that the LGBTQ+ community face, both during the pandemic and beyond.

### 3.4. Families in a Time of COVID-19

As a result of the COVID-19 outbreak 1.38 billion children are out of school or childcare, and unfortunately, the burden of this falls disproportionately on women. Reported rates of child abuse increase during school closures, and are likely to increase in times of uncertainty and increased stress. This is in turn likely to be more severe in already crowded households and those under increased economic pressure. Large non-governmental bodies have released open-access online parenting resources, focusing on concrete advice on how to build positive relationships, divert and manage disruptive behavior, and manage parenting stress. They are available through social media and through non-smart phones through the *Internet of Good Things* [42].

The impact of the COVID-19 on families was studied in a population based survey of perceived harms and benefits of the pandemic in Hong Kong. In total, 19.0% of respondents perceived a benefit for family physical health, with a corresponding 7.2% for family mental health, and 13.5% for family relationships. The harms, however, outweigh the benefits, with 37.9% anticipating a deterioration in family mental health and 18.6% in family relationships [43]. In light of these findings, it is important that policymakers provide urgent assistance, particularly to vulnerable families, who are likely to be disproportionally impacted by the pandemic.

Subjective perceptions of harm, feelings of powerlessness, and chronic stress may contribute to the increased prevalence of psychiatric symptoms in the general population. Parents of children who have tested positive for COVID-19 are more likely to display features of post-traumatic stress, anxiety, and depression, with mothers particularly at risk [44].

### 3.5. Risk Perception

People’s willingness to accept government imposed non-pharmaceutical interventions is intrinsically linked to public risk perception. This theme was explored by a team from Cambridge University, who began by gathering data and plotting mean risk perceptions scores. Unsurprisingly, the countries with the highest exposure rates (UK and Spain) also perceived the highest levels of risk. In addition to population level analysis, individual risk perception was correlated to personally held values. An increased risk perception was associated with a direct experience of the virus, people who believe that it is important to do things for the benefit of others and society more broadly, those who trust in science, and those suspicious of the government. The inverse was also true; the more individualistic the character, the lower the perception of risk [45]. 

Related work was conducted in Mexico City, concerning perception of severity and education levels in older people [46]. Unlike in the Cambridge study, where males generally perceived lower risk than females, no significant gender differences were found. Equally, the source of information did not appear to influence risk perception, although a majority (67.6%) of older people received the bulk of their knowledge from the television. The authors did however find a significant association between income levels and perceived risk: with those on low incomes three times less likely to stay at home, compared to those on middle incomes. High levels of educational attainment were similarly correlated with increased risk perception and risk reduction behavior [46].

The results of both of these studies reinforce the difficulties governments face in influencing risk perception, which is often based on fixed societal factors, as well as intrenched cultural values. This is particularly true in serious psychiatric illnesses, including schizophrenia spectrum disorders, where disorders of cognition and judgement constitute part of the clinical syndrome [47].

## 4. Conclusions

Non-pharmaceutical interventions to reduce transmission, infections, morbidity, and death seem to have been effective in limiting the scale and duration of the first and second waves of COVID-19. The consequences, however, of social isolation, stay-at-home measures, lockdowns, physical distancing, and other containment strategies, as well as the resulting economic downturn, have led to exacerbations of health inequalities and an increase in adverse mental health effects [48]. The burden of illness falls both on those who have never previously had to access psychiatric care, as well as those known to mental health services. Health disparities, both globally and within individual countries, have been highlighted by the pandemic. Many of these disparities have mental health consequences. Mental health budgets globally remain stubbornly low, and following the economic challenges of the COVID-19 response, this is unlikely to change. It is important for mental health practitioners to be aware of the risk of promoting short-term, cheap solutions to broaden access to mental health. Treatment based on affordability rather than efficacy or need is likely to exacerbate the inequalities and impoverish mental health more broadly [48].

With the introductions of mass vaccination programs, governments are beginning to look forward to a post-COVID-19 world. Whilst this is unlikely ever to become a reality, mental health practitioners can also use this moment to reflect on how our services should develop. The medical roles of treatment and prevention can be extrapolated to a population level. Within the scope of this subject, treatment constitutes the provision, maintenance, and improvement of existing services; prevention involves advocating for vulnerable communities, both with and without pre-existing psychiatric conditions. By increasing awareness of the impact of the pandemic on specific groups, individualized plans of treatment and prevention can be introduced to improve mental health care for all.

## Data Availability

Data sharing not applicable to this article as no datasets were generated or analyzed during the current study.
